# Blau syndrome: Lessons learned in a tertiary care centre at Chandigarh, North India

**DOI:** 10.3389/fimmu.2022.932919

**Published:** 2022-09-15

**Authors:** Rajni Kumrah, Rakesh Kumar Pilania, Nitin Kumar Menia, Amit Rawat, Jyoti Sharma, Anju Gupta, Pandiarajan Vignesh, Ankur Kumar Jindal, Rashmi Rikhi, Aniruddha Agarwal, Vishali Gupta, Surjit Singh, Deepti Suri

**Affiliations:** ^1^ Pediatric Allergy Immunology Unit, Advanced Pediatrics Centre, Postgraduate Institute of Medical Education and Research, Chandigarh, India; ^2^ Department of Ophthalmology, Advanced Eye Centre, Postgraduate Institute of Medical Education and Research, Chandigarh, India

**Keywords:** arthritis, blau syndrome, uveitis, granulomatous inflammation, NOD2 mutation

## Abstract

**Objectives:**

Blau syndrome (BS) is a rare autoinflammatory disease characterized by arthritis, dermatitis, and granulomatous uveitis in early childhood. The study presents the clinical experience of patients with BS at a tertiary care centre in Chandigarh, North India.

**Methods:**

Analysis of the clinical profile of patients of BS with *NOD2* gene mutations under follow-up was carried out.

**Results:**

Diagnosis of BS was genetically confirmed in 11 patients (10 children and one adult; six male and five female patients) from 10 families. The median age of onset of symptoms was 12 months (range, 4 months–4 years), while the age at diagnosis ranged from 2.3 to 26 years. The classic triad of arthritis, dermatitis, and uveitis was present in 6/11 (54.5%) patients. The frequency of arthritis, dermatitis, and uveitis was 100%, 81.8%, and 72.7%, respectively. The median age at diagnosis of ocular symptoms was 4 years (range, 2–26 years). Family history was noted in six families. Renal involvement was observed in two children. All patients in our cohort had the R334W variant in *NOD2* gene. An asymptomatic carrier sibling with R334W mutation was identified in one family. Methotrexate was used as a first-line agent in all children. Adalimumab, which was commenced in five patients with uveitis, resulted in significant improvement in four patients. The total follow-up duration of the present cohort is 1,063.8 patient-months.

**Conclusions:**

The possibility of BS should always be considered in patients with arthritis and early ocular involvement. Uveitis is often progressive and refractory to currently available therapies. Systemic involvement appears to remain a significant cause of morbidity and mortality.

## Introduction

Blau syndrome (BS) is a rare monogenic form of the autoinflammatory disease (AIDs) caused by a gain-of-function mutation in the *Caspase Recruitment Domain 15* (*CARD 15*)/*Nucleotide Oligomerization Domain of 2* (*NOD2*) gene. It is characterized by a triad of arthritis, dermatitis, and granulomatous uveitis in early childhood (1, 2). In his initial description, Dr. Edward Blau reported that 11 members of a single family over four generations had developed granulomatous disease of the joints, skin, and eyes (3). Later, Jabs et al. described a family with a syndrome of granulomatous synovitis, recurrent uveitis, and cranial neuropathies (4). Several systemic associations have since been described in the context of BS (4–8). A molecular defect responsible for BS was identified in 2001, and mutations in the nucleotide oligomerization domain in *CARD15/NOD2* gene located on chromosomal region 16q12.1-13 were mapped (9). Diagnosis of BS is based on classical clinical features, family history, and demonstration of non-caseating granulomas and can be confirmed by genetic analysis.

Ben-Chetri et al. (10) have proposed the definitions and consensus nomenclature for the different AIDs. As per this classification BS has been categorized under the subheading of NOD2-associated granulomatous disease. Other than BS, familial sarcoidosis, and familial Crohn’s disease have also been classified under this subheading.

There is a lack of information on this disease from the Indian subcontinent (8, 11–14). This study reports clinical experience while managing 11 patients from nine families with BS at a tertiary care centre in North India.

## Patients and methods

Case records of patients attending the Pediatric Rheumatology Clinic, Advanced Pediatrics Centre, Postgraduate Institute of Medical Education and Research, Chandigarh, India, were reviewed. A detailed analysis of the clinical profile, investigations, treatment, and outcome of children with BS was recorded. An ophthalmological examination of these children was carried out at the uveitis clinic, Advanced Eye Centre, PGIMER, Chandigarh. The genetic diagnosis was confirmed at Pediatric Immunology Laboratory, PGIMER, Chandigarh.

Peripheral blood samples were collected for molecular analysis after obtaining informed consent. Exon-4 of *NOD2* gene was amplified using polymerase chain reaction (PCR) at controlled conditions using specific oligonucleotide primers, which were obtained from the Resource of Asian Primary Immunodeficiency Database (RAPID) (15). The PCR products were qualitatively examined by 1.5% agarose gel electrophoresis followed by purification and direct sequencing using the ABI Big Dye Terminator kit and ABI 3500 Gene Analyzer (Applied Biosystems, Foster City, CA, USA). Sequencing results were analysed using Codon Code Aligner software (Codon Code Corporation, Centerville, MA, USA).

## Results

### Demographic characteristics

BS was genetically confirmed in 11 patients (10 children and one adult; six male and five female patients) from 10 families. The mean age of onset of symptoms was 17.3 ± 14.6 months (median, 12 months; range, 4 months–4 years), while the median age at presentation to our institute was 2 years 10 months (range, 10 months–26 years). Delay in diagnosis from onset of symptoms ranged from 10 months to 20 years. The median age at diagnosis in our cohort was 7.9 years (range, 2.3 – 26 years).

### Clinical characteristics

The clinical possibility of BS was considered on 5/11 (45.5%) at presentation to our institute. In the remaining six patients (54.5%), a BS diagnosis was made later at follow-up. The latter patients were being followed up for variable periods as polyarticular juvenile idiopathic arthritis (JIA) (n = 3), systemic JIA (n = 1), Poncet’s disease (n = 1), and early-onset sarcoidosis (n = 1 adult) (**Table 1**).

**Table 1:** Clinical features, laboratory investigations, and treatment details of patients with Blau syndrome.

Patient no.	Age at onset (years)/sex	Age at presentation (years)	Age at diagnosis(years)	Delay in diagnosis from onset of symptoms (years)	Initial diagnosis	Articular manifestations	Rash	Uveitis	Age at diagnosis of uveitis and initiation of treatment for uveitis (years)	Other features	Family history	Hb (g/L)	TLC (×10^9^/L)	Platelet count (×10^9^/L)	ESR (mm/hr)	CRP (mg/L)	Follow-up duration (years)	Drugs received
1	0.5/M	2.9	2.9	2.3	BS	Boggy swelling, tenosynovitis, camptodactyly, polyarthritis (wrist, ankle, small joints of hands)	Yes	Chronic anterior uveitis with superficial corneal opacity	2	No	No	96	8.1	562	32	12	2.11	MTX, PRED
2	2/F	2.10	2.10	0.8	BS	Boggy swelling, camptodactyly, polyarthritis (wrist, ankle, small joints of hands)	Yes	Bilateral anterior uveitis	2	Fever	Yes, affected father	104	12.5	644	67	38.2	3.7	MTX, PRED
3	0.4/M	0.8	9.11	9.5	*SJIA*	Boggy swelling, camptodactyly, polyarthritis (wrist, ankle, small joints of hands and knees)	No	Panuveitis; nebular corneal opacities at 10 monthsBilateral uveitis at 4 years; acute angle closure glaucoma and cataract; surgery for glaucoma and cataract at 15 years of age	10 months	Fever	Yes affected mother (pedigree family 1 in **Figure 2**)	80	15.2	377	44	20	14.6	MTX, PRED, AZA, MMF
4 (cousin of pt. 3)	3/F	11.3	20.5	17.5	PolyJIA	Multiple joint deformities, tenosynovitis, polyarthritis (wrist, ankle, small joints of hands)	No	Panuveitis, bilateral uveitis, posterior synechiae, bilateral vitritis, retinal vascular leakage, glaucoma at 11 years, bilateral cataract at 13 years of age, surgery for cataract	10	Fever	Yes affected father (pedigree family 1 in **Figure 2**)	112	8.0	210	50	11.4	9.11	MTX, PRED, AZA, adalimumab (40 mg every 2 weeks)
5	1/F	3	21	20	Poncet’s disease	Boggy swelling, camptodactyly, polyarthritis (wrist, ankle, small joints of hands and knees)	No	Panuveitis, bilateral uveitis, posterior synechiae	13	Fever, visceral involvement (liver, kidney)	No	112	10.6	294	12	1.87	19.8	MTX, PRED, MMF, adalimumab (40 mg every 2 weeks)
6	0.3/M	2.4	14.3	14	PolyJIA	Boggy swelling, camptodactyly, polyarthritis (wrist, ankle, small joints of hands)	Yes	Bilateral recurrent panuveitis with corneal opacities, hypopyon	2.4	No	No	115	6.4	327	20	10	12.4	MTX, AZA, leflunomide, adalimumab (40 mg every 2 weeks)
7	4/M	6.11	6.9	2.9	BS	Boggy swelling, tenosynovitis, camptodactyly, polyarthritis (wrist, ankle, small joints of hands, knees, and shoulders)	Yes	Panuveitis, bilateral uveitis. cataract surgery for both eyes at 11 years of age had right eye vision loss due to total retinal detachment and phthisical eye, at 13 years of age	7	Visceral involvement (kidney), tuberculosis meningitis	Yes affected mother (pedigree family 2 in **Figure 2**)	81	10.1	398	14	31.56	8.2	MTX PRED, AZA, adalimumab (40 mg every 2 weeks)
8 (sibling of pt. 7)	1/F	8.11	8.9	7.9	BS	Tenosynovitis, camptodactyly, polyarthritis (wrist, ankle, small joints of hands, knees, and hips)	Yes	No eye involvement	No eye involvement	No	Yes affected mother (pedigree family 2 in **Figure 2**)	115	9.9	228	32	28.98	8.1	MTX, PRED, AZA
9	1.6/F	2.6	5.2	3.8	*PolyJIA*	Boggy swelling, camptodactyly, polyarthritis (wrist and ankle)	Yes	Panuveitis, bilateral chronic uveitis, secondary bilateral cataract, multifocal choroiditis	2 months	No	Yes affected father with nodular skin lesions	100	8.2	421	40	15	7.7	MTX, adalimumab (40 mg every 2 weeks)
10	0.6/M	2.4	2.3	1.7	BS	Boggy swelling, tenosynovitis, polyarthritis (wrist and ankle)	Yes	No eye involvement	No eye involvement	Fever, granulomatous lymphadenopathy	Yes affected mother (pedigree family 3 in **Figure 2**)	111	10.2	524	22	8.52	0.5	MTX, PRED
11	5/M	26	26	21	BS	Boggy swelling and polyarthritis (wrist, ankle, small joints of hands and knees)	Yes	Bilateral chronic anterior uveitis with sequelae, right eye cataract	26	No	No	111	6.2	600	20	19	6 days	MTX

BS, Blau syndrome; CRP, C-reactive protein; ESR, erythrocyte sedimentation rate; Hb, haemoglobin; PolyJIA, polyarticular juvenile idiopathic arthritis; SJIA, systemic juvenile idiopathic arthritis; EOS, early-onset sarcoidosis; MTX, methotrexate; PRED, prednisolone; AZA, azathioprine; MMF, mycophenolate mofetil; TLC, total leucocyte count.

**Figure 1:**
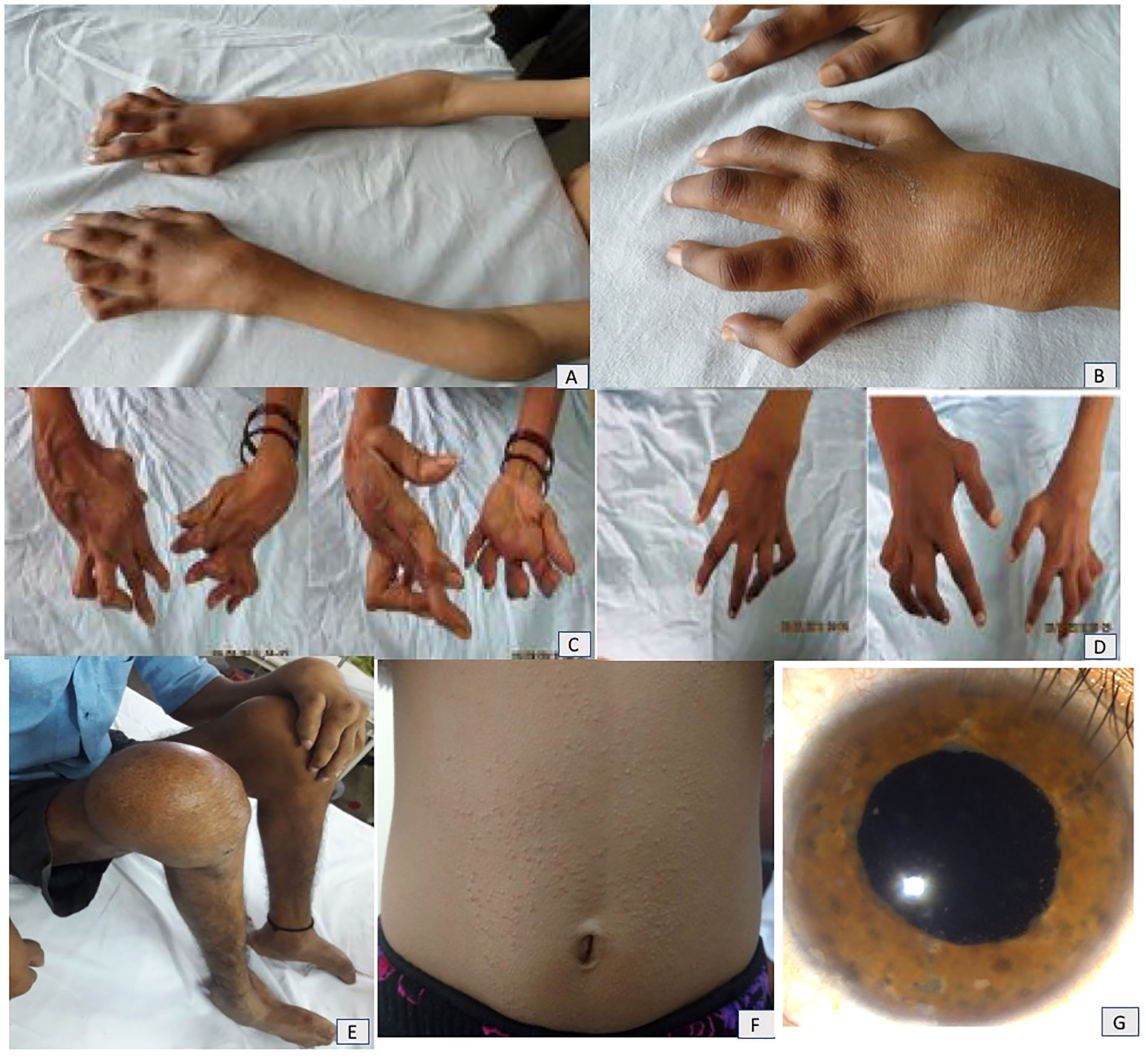
**(A, B)** Boggy swelling of wrist, elbow, and small joints of hands and camptodactyly in patient no. 7. Mother **(C)** and elder sister **(D)** of patient no. 7 showing extreme deformities of small joints of hands. **(E)** Residual joint deformities of bilateral foot and knee as well as small joints of hands in patient no. 11. **(F)** Micropapular rash on trunk in patient no. 10. **(G)** Anterior segment photograph of a patient with Blau syndrome shows presence of iris nodules, anterior chamber cells, and flare suggestive of chronic anterior uveitis.

A classic triad of arthritis, dermatitis, and uveitis was present in 6/11 (54.5%) children. All patients had wrist and ankle involvement. The characteristic “boggy” swelling of the joints was noted in nine (81.8%). Tenosynovitis of tendons resulting in contractures, deformities of the small joints, and erosive joints was seen in five (45%) patients (patient nos. 1, 4, 7, 8, and 10)—these patients had had significant delays in diagnosis. Camptodactyly was observed in seven (63.6%) patients. A history of micropapular rash was noted in eight (73%) patients. It was reported to be milder and improved at follow-up (**Figure 1**).

Uveitis was seen in nine (81.8%) patients, and two patients (patient nos. 8 and 10) have not developed any ocular involvement so far. The median age at diagnosis of uveitis was 4 years (range, 2–26 years). Uveitis was noted in seven patients at first presentation, while two patients (patient nos. 3 and 5) developed uveitis at follow-up. Both these patients were being followed up for arthritis, and uveitis developed later in the course after 3 and 10 years of follow-up. All nine patients had bilateral eye involvement (18 eyes), while 12 eyes had panuveitis, and six had anterior uveitis. Anterior chamber cells and flare were present in all patients with uveitis. Posterior synechiae were seen in 17 eyes. Granulomatous keratic precipitates (KPs) were seen in eight eyes, non-granulomatous KPs in two eyes, and iris nodules in two eyes. Other features including vitritis (six eyes), multifocal choroiditis (12 eyes), retinal vasculitis (two eyes), optic nerve head oedema (one eye), and cystoid macular oedema (one eye) were also present. Complications were present in form of band-shaped keratopathy (seven eyes), nebular coronal opacities (one eye), complicated cataract (12 eyes), iris bombe (two eyes), secondary glaucoma (seven eyes), and phthisis bulbi (two eyes). There were multiple episodes of recurrences despite immunosuppressive therapies **(** **Table 1**).

Visceral involvement was seen at follow-up in two patients (patient nos. 5 and 7). Patient no. 5 developed hepatosplenomegaly, ascites, portal hypertension, nephrotic range proteinuria, and microscopic haematuria at 20 years of age. Liver and renal biopsies showed granulomatous disease, which helped establish the diagnosis and has been previously reported (8). Patient no. 7 developed chronic kidney disease with worsening renal function and nephrotic range proteinuria at 11 years of age. Renal biopsy demonstrated granulomatous interstitial nephritis with progressive glomerulosclerosis. He was initiated on adalimumab (40 mg subcutaneously every 2 weeks) for refractory disease to conventional disease-modifying antirheumatic drugs (DMARDs). However, he developed tubercular meningitis and succumbed to his illness.

**Figure 2:**
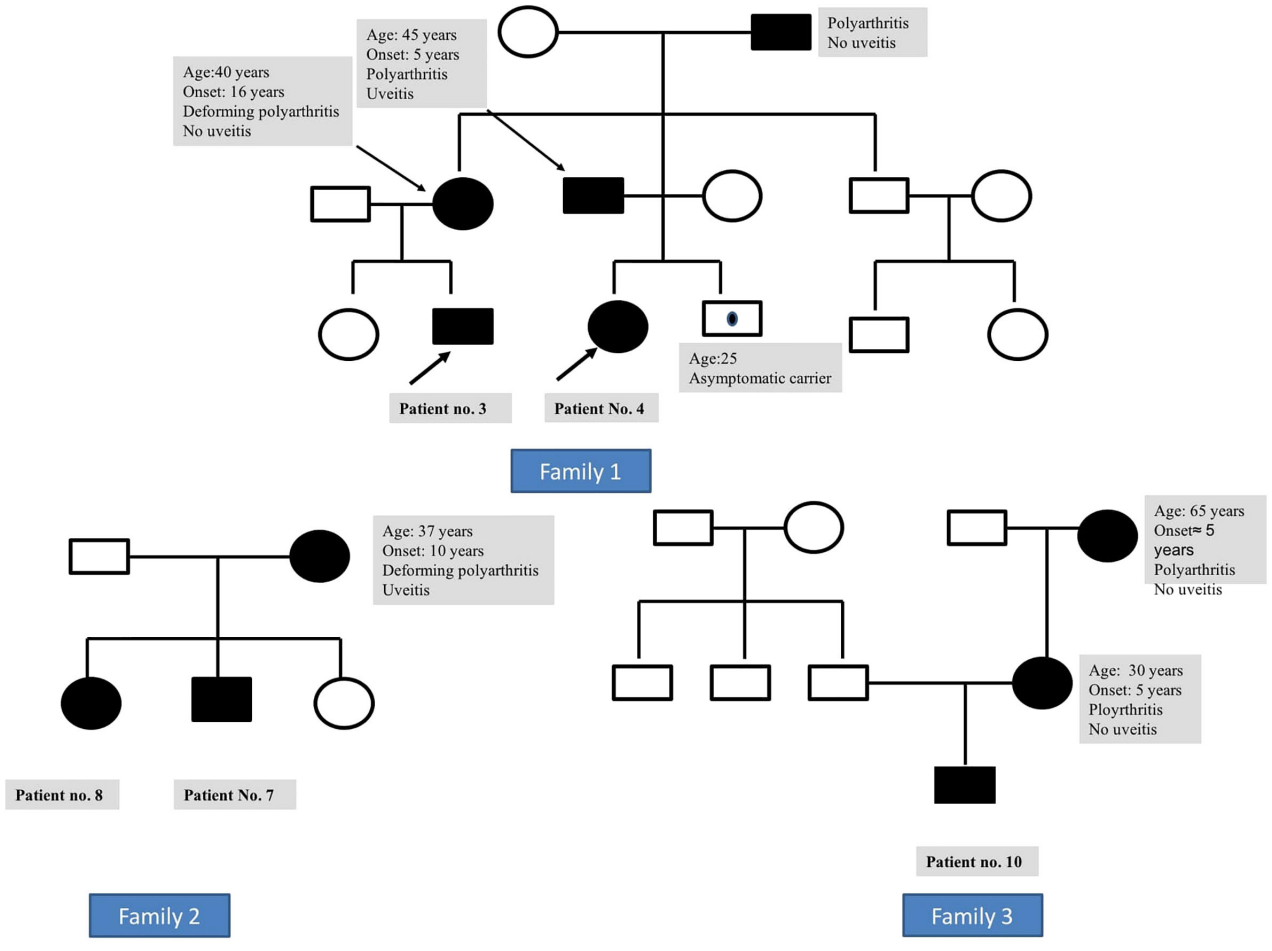
Family 1: pedigree of patient nos. 3 and 4 (siblings) showing disease manifestations in affected individuals with Blau syndrome. Family 2: pedigree of patient nos. 7 and 8 showing disease manifestations in affected individuals with Blau syndrome. Family 3: pedigree of patient no. 10 showing disease manifestations in affected individuals with Blau syndrome.

The family history of an affected parent was available in six families (three mothers and three fathers), while the disease was sporadic in four patients (**Figure 2**). Symptomatic parents had arthritis (n = 5), uveitis (n = 2), or both (n = 2). None of these parents recalled having had a rash during childhood. Nodular skin lesions without arthritis or uveitis were noted in the father of patient no. 9.

### Laboratory features

Inflammatory markers, erythrocyte sedimentation rate (ESR), and C-reactive protein (CRP) were elevated in all patients at first presentation. Platelet counts ranged from 2.4 × 10^9^ to 8 × 10^9^/L. Fine-needle aspirations of boggy joint swelling were performed in five patients; however, granulomatous inflammation could be seen in one patient only. Two patients underwent skin and synovial biopsies that revealed granulomatous inflammation.

### Genetic analysis

**Figure 3:**
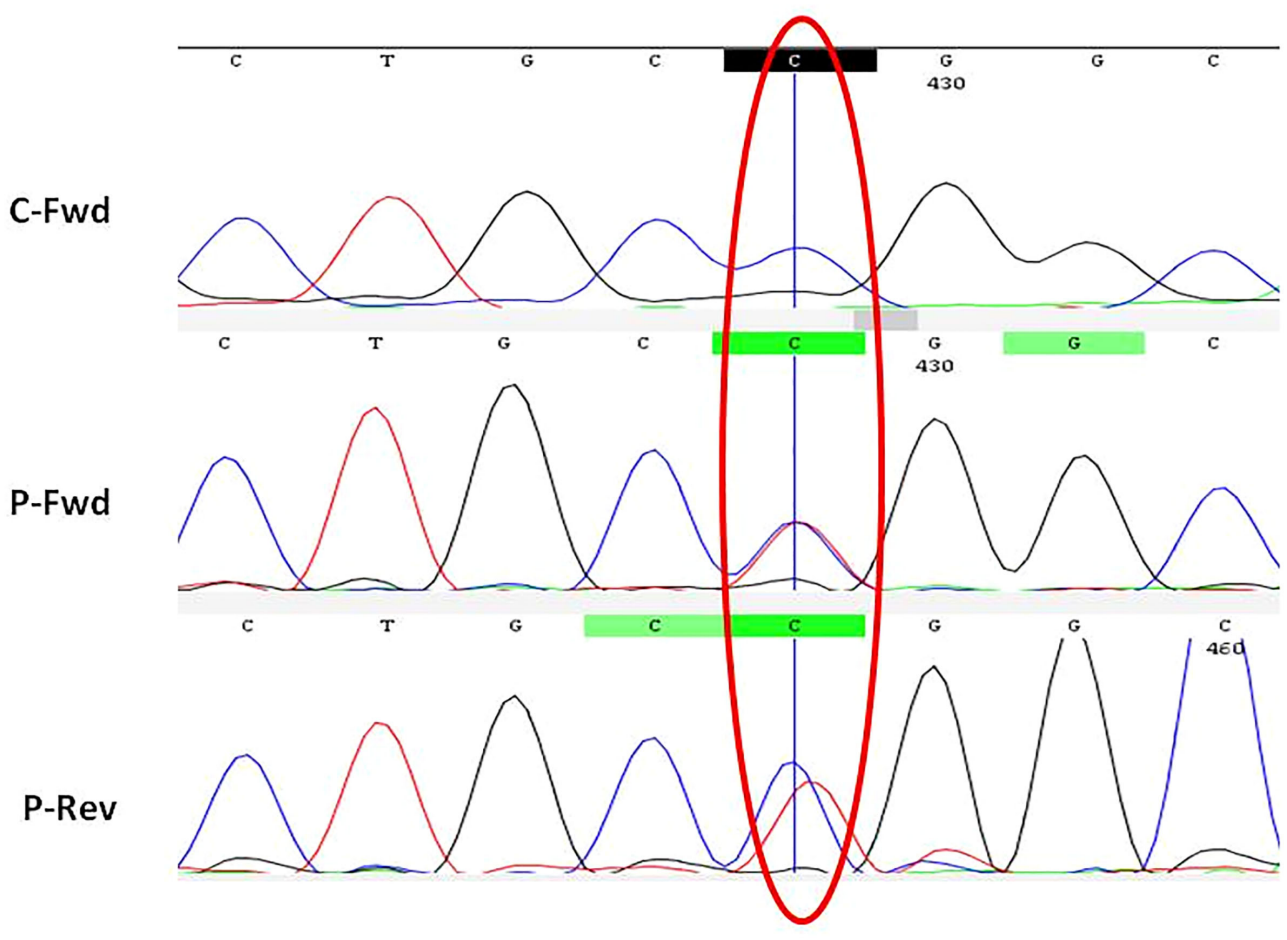
Electropherogram showing *NOD2* gene (Exon-4) with missense heterozygous variant (p.R334W). C, control; P, patient; Fwd, forward; Rev, reverse.

A missense heterozygous variant causing an amino acid change from arginine to tryptophan at position 334 (hotspot) was identified in the nucleotide-binding domain of *NOD2* gene in all patients (**Figure 3**). Affected symptomatic parents were also found to have the same mutation. The mutation was *de novo* in four children. The brother of patient no. 4 was asymptomatic but carried the same mutation. He has remained well and is currently 25 years old.

### Treatment details

Subcutaneous weekly methotrexate (15–20 mg/m^2^/week) was used as a first-line agent for arthritis and uveitis. Oral prednisolone was used intermittently for disease flares, in addition to topical ocular steroids and atropine drops. Seven (63.6%) patients required additional therapy for uveitis refractory to conventional DMARDs. These included azathioprine (n = 6), mycophenolate mofetil (n = 3), and leflunomide (n = 1). Five patients were initiated on adalimumab (40 mg subcutaneously every 2 weekly)—four had significant improvement in visual acuity and inflammation, while adalimumab had to be discontinued in one patient due to the reactivation of tuberculosis **(** **Table 1**).

### Outcome and follow-up details

All children continued to be symptomatic at follow-up and did not achieved drug-free remission. The present median age at the latest follow-up is 15 years (5.7–26 years). The mean duration of follow-up is 1,063.8 patient-months. Residual joint deformities limiting daily activities were seen in four children. Uveitis was in remission in six patients, while three patients continued to have active disease. Renal involvement was observed in two children. One of these two patients developed reactivation of tuberculosis leading to tubercular meningitis while on adalimumab therapy and succumbed to his illness.

## Discussion

BS is a rare genetic cause of early-onset arthritis and uveitis in children with autosomal dominant inheritance (1). In addition to classical clinical features, non-caseating epithelioid and giant cell granulomas in affected tissues are the hallmark. A timely and correct diagnosis is essential, as it has several implications regarding prognosis, treatment, long-term follow-up, and genetic counselling.

Although BS was described three decades ago and the genetic basis was identified in 2001, it remains an under-recognized entity in clinical practice (3, 4, 9). The available literature on BS emanates from developed countries. Small case studies have been recently reported from India (8, 11, 12). There are limited data on the clinical profile, molecular diagnosis, and long-term outcome of patients with BS, especially from the developing world. We herein report a case series of genetically confirmed patients of BS from North India, highlighting the disease’s evolution, catastrophes due to delays in diagnosis, and treatment options with follow-up.

BS is characterized by a triad of rash, arthritis, and uveitis. The rash is usually the first to appear (first year of life), followed by arthritis (2–4 years of age), and, lastly, uveitis (around 4 years of age) (2). Complete triad was present in only 55% of patients in our cohort at the time of presentation.

Fever has not been included in the classic triad but is a particularly important clinical manifestation in young children. Matsuda et al. have recently reported that approximately 50% of patients had a fever since the early course of the illness (16). Skin involvement (maculopapular or micropapular scaly rash) occurs during infancy and often precedes joint involvement and ocular symptoms. The presence of rash and fever with markedly raised inflammatory parameters often results in diagnostic dilemmas, with systemic juvenile idiopathic arthritis (sJIA) being the closest differential. Rash in BS compared to sJIA is not evanescent but erythematous and fixed or scaly. Aroestegui et al. reported 12 patients with BS, of whom 50% had sJIA as the initial diagnosis. All these children had presented before 4 years of age (17). History of early-onset rash was present in eight (72.7%) patients in our cohort, similar to published literature (18), however, it was not persistent. An infant (patient no. 3) was diagnosed with sJIA because of fever, rash, arthritis, and raised inflammatory parameters. Development of uveitis later in the course of the disease resulted in the revision of the diagnosis. Uveitis is distinctly unusual in patients with sJIA. Therefore, it is essential to highlight that BS must always be considered a possibility if children with sJIA develop uveitis at follow-up.

Also, BS must always be considered a differential in children presenting with polyarticular symptoms. Polyarthritis involving the peripheral joints (wrist, ankle, knee, and small joints of the hands and feet) and typical “boggy” non-tender swelling with synovial hypertrophy are observed. Typically, large joint arthritis is non-erosive, associated with little movement restriction, and resolves without deformities, though erosive arthritis has also been reported (19) (1, 2). Exuberant tenosynovitis in BS leads to flexion contractures of the interphalangeal joints, camptodactyly, and characteristic deformities of the hands and feet. Arthritis was the most dominant complaint in our patient population also. Erosive arthritis was seen in 5/11 patients. The presence of boggy swelling, camptodactyly, and bilaterally symmetrical arthritis involving the wrist and small joints of the hands were the most consistent clinical clues to the diagnosis of BS (1, 8, 16).

The most common ocular manifestations of BS include bilateral recurrent uveitis. However, keratoconjunctivitis sicca, conjunctival granulomas, optic nerve involvement, band-shaped keratopathy, cataract, glaucoma, and rarely subepithelial corneal opacities have also been described (1, 16). Cataract and glaucoma in these children may be related to both chronic ongoing uveitis and long-term corticosteroid therapy. The presence of uveitis is the most important clue to the diagnosis of BS, but incomplete phenotypes are not uncommon. Approximately 10%–20% of patients may not develop eye disease (1, 16). Uveitis was not seen in two children and seven parents who carried the mutation.

Chronic posterior uveitis is the most common finding, but it can evolve into multifocal choroiditis and panuveitis. Uveitis is often recurrent, treatment-refractory, and blinding. It remains the most important cause of morbidity in patients with BS and requires aggressive screening and treatment for a better quality of life for these patients. Commonly used drugs for BS-associated uveitis include systemic corticosteroids, methotrexate, azathioprine, mycophenolate mofetil, and tumour necrosis factor (TNF) blockers. Published literature has shown favourable experience with anti-TNF agents in the management of uveitis associated with BS (8, 16, 20). Sarens et al. carried out a multi-centric, prospective, interventional study in patients with BS-associated uveitis. They showed that despite aggressive local and additional systemic therapies, ocular disease activity is insufficiently controlled in most patients (20). All our patients received subcutaneous methotrexate therapy as initial therapy. Almost two-thirds (63.6%) of patients required additional therapeutic agents. Adalimumab was useful in four patients to achieve remission but had to be discontinued in one due to reactivation of tuberculosis.

Systemic and visceral involvement in patients with BS has been described, such as granulomas in the liver, intestines, parotid glands, lymph nodes, and kidney; tubulointerstitial nephritis; cutaneous ulcers; and granulomatous arteritis (8, 16, 21). Renal involvement occurred in two patients and was the cause of death in one child who developed as early as 11 years of age.

Miceli-Richard et al. identified *NOD2* as the causative gene in BS and described three different single base-pair mutations in four families in 2001 (9). In the last two decades, several NOD2 mutations have been described in association with BS. Most mutations reported to date are at or near the nucleotide-binding NOD/NACHT domain of *NOD2* gene, and some are also found to extend into the C-terminal region characterized by a leucine-rich repeat (LRR) structure (1, 2). The most common mutation reported is the substitution of arginine at position 334 by glutamine (R334Q) or tryptophan (R334W) (1, 16). In the present study, all patients had R334W mutation. Although the penetrance of Blau-associated NOD2 mutations is very high, Saulsbury et al. reported an asymptomatic carrier of E383K substitution (22). We identified one asymptomatic carrier with R334W substitution. To the best of our knowledge, this is the first report of an asymptomatic carrier of BS with the R334W variant.

To conclude, the possibility of BS should always be considered in patients with polyarthritis and early ocular involvement, especially if family history is suggestive. Patients may not demonstrate the classic triad at the time of initial presentation. Fever is an important manifestation in young children. Patients are often misdiagnosed as sJIA or polyarticular JIA. Polyarthritis with synovial hypertrophy and tendon contractures resulting in deformities of the hands are clues to diagnosis. Uveitis in patients with BS is severe and usually refractory to conventional DMARDs. However, TNF blockers have shown promising results in these patients. Systemic involvement remains an important cause of morbidity and mortality. Hotspot screening for NOD2 mutation can be incorporated as a screening tool while evaluating patients with uveitis for early diagnosis of this disease. More efforts and awareness are necessary amongst paediatric rheumatologists about the presentation of BS to avoid significant delays in diagnosis.

## Key messages

- Blau syndrome is a rare autoinflammatory disease caused by a gain-of-function mutation in *NOD2* gene.
- A complete triad of arthritis, uveitis, and dermatitis may not be present at the time of presentation.
- Fever is an important manifestation in young children.
- Polyarthritis, boggy synovitis, camptodactyly, and deformities of the small joints of the hands are important clues.
- Uveitis is difficult to treat and often requires TNF blockers.
- R334W is the commonest mutation in the Indian population.
- Hotspot screening for NOD2 mutation can be incorporated as a screening tool while evaluating patients with uveitis for early diagnosis of BS.
- Renal involvement is an important cause of mortality.

## Think of Blau syndrome

- In infants with fever and fixed rash
- In young children with fever, rash, and uveitis
- In patients with polyarticular arthritis with uveitis
- In patients with arthritis and a family history
- In patients with treatment-refractory bilateral uveitis
- In patients developing contractures of the small joints of the hands and camptodactyly
- In patients with non-caseating granulomas in tissues

## Data availability statement

The original contributions presented in the study are included in the article/supplementary material. Further inquiries can be directed to the corresponding author.

## Ethics statement

This study was reviewed and approved by Institute Ethics Committee, PGIMER, Chandigarh. Written informed consent to participate in this study was provided by the participants’ legal guardian/next of kin.

## Author contributions

RK, RP, AR, and DS designed the research. RK, RP, NM, AR, JS, AG, PV, AJ, RR, AA, VG, GD, SS, and DS performed the research. RK, RP, NM, DS, and SS wrote the paper. RK, RR, JS, and AR performed the genetic analysis of these patients. NM, AA, and VG performed the ophthalmological examination. DS, AR, and SS critically supervised the manuscript. All authors contributed to the article and approved the submitted version.

## Funding

The authors thankfully acknowledge the Post Graduate Institute of Medical Education and Research Chandigarh India for funding under Short Term Research Scheme vide sanction no 71/2-EDU-16/585, Project ID: 7221.

## Acknowledgments

We sincerely thank the patients and their parents for their cooperation.

## Conflict of interest

The authors declare that the research was conducted in the absence of any commercial or financial relationships that could be construed as a potential conflict of interest.

## Publisher’s note

All claims expressed in this article are solely those of the authors and do not necessarily represent those of their affiliated organizations, or those of the publisher, the editors and the reviewers. Any product that may be evaluated in this article, or claim that may be made by its manufacturer, is not guaranteed or endorsed by the publisher.

## References

[1] Wouters CH Maes A Foley KP Bertin J Rose CD. Blau syndrome, the prototypic auto-inflammatory granulomatous disease. Pediatr Rheumatol Online J (2014) 12:33. doi: 10.1186/1546-0096-12-33 25136265 PMC4136643

[2] Rosé CD Wouters CH Meiorin S Doyle TM Davey MP Rosenbaum JT. Pediatric granulomatous arthritis: an international registry. Arthritis Rheum (2006) 54(10):3337–44. doi: 10.1002/art.22122 17009307

[3] Blau EB. Familial granulomatous arthritis, iritis, and rash. J Pediatr (1985) 107(5):689–93. doi: 10.1016/S0022-3476(85)80394-2 4056967

[4] Jabs DA Houk JL Bias WB Arnett FC. Familial granulomatous synovitis, uveitis, and cranial neuropathies. Am J Med (1985) 78(5):801–4. doi: 10.1016/0002-9343(85)90286-4 3993660

[5] Meiorin SM Espada G Costa CE Tartara A De Matteo E Wouters C. Granulomatous nephritis associated with R334Q mutation in NOD2. J Rheumatol (2007) 34(9):1945–7. 17787056

[6] Wang X Kuivaniemi H Bonavita G Mutkus L Mau U Blau E. CARD15 mutations in familial granulomatosis syndromes: a study of the original blau syndrome kindred and other families with large-vessel arteritis and cranial neuropathy. Arthritis Rheum (2002) 46(11):3041–5. doi: 10.1002/art.10618 12428248

[7] Ting SS Ziegler J Fischer E. Familial granulomatous arthritis (Blau syndrome) with granulomatous renal lesions. J Pediatr (1998) 133(3):450–2. doi: 10.1016/S0022-3476(98)70286-0 9738733

[8] Jindal AK Pilania RK Suri D Gupta A Gattorno M Ceccherini I. A young female with early onset arthritis, uveitis, hepatic, and renal granulomas: a clinical tryst with blau syndrome over 20 years and case-based review. Rheumatol Int (2021) 41(1):173–81. doi: 10.1007/s00296-019-04316-6 31062074

[9] Miceli-Richard C Lesage S Rybojad M Prieur AM Manouvrier-Hanu S Häfner R. CARD15 mutations in blau syndrome. Nat Genet (2001) 29(1):19–20. doi: 10.1038/ng720 11528384

[10] Ben-Chetrit E Gattorno M Gul A Kastner DL Lachmann HJ Touitou I. Paediatric Rheumatology International Trials Organisation (PRINTO) and the AIDs Delphi study participants. Consensus proposal for taxonomy and definition of the autoinflammatory diseases (AIDs): a Delphi study. Ann Rheum Dis (2018) 77(11):1558–8. doi: 10.1136/annrheumdis-2017-212515 30100561

[11] Janarthanan M Poddar C Sudharshan S Seabra L Crow YJ. Familial blau syndrome:First molecularly confirmed report from India. Indian J Ophthalmol (2019) 67(1):165–7. doi: 10.4103/ijo.IJO_671_18 PMC6324106 30574935

[12] Babu K Rao AP. Clinical profile in genetically proven blau syndrome: A case series from south India. Ocul Immunol Inflamm (2020), 29(2):250–6. doi: 10.1080/09273948.2020.1746353 32293936

[13] Jindal AK Pilania RK Rawat A Singh S. Primary immunodeficiency disorders in India-a situational review. Front Immunol (2017) 8:714. doi: 10.3389/fimmu.2017.00714 28674536 PMC5474457

[14] Pilania RK Chaudhary H Jindal AK Rawat A Singh S. Current status and prospects of primary immunodeficiency diseases in Asia. Genes Dis (2020) 7(1):3–11. doi: 10.1016/j.gendis.2019.09.004 32181271 PMC7063407

[15] Resource of Asian primary immunodeficiency disease. Available at: http://web16.kazusa.or.jp/rapid/lite.cgi.

[16] Matsuda T Kambe N Ueki Y Kanazawa N Izawa K Honda Y. Clinical characteristics and treatment of 50 cases of blau syndrome in Japan confirmed by genetic analysis of the NOD2 mutation. Ann Rheum Dis (2020) 79(11):1492–9. doi: 10.1136/annrheumdis-2020-217320 32647028

[17] Aróstegui JI Arnal C Merino R Modesto C Antonia Carballo M Moreno P. NOD2 gene-associated pediatric granulomatous arthritis: clinical diversity, novel and recurrent mutations, and evidence of clinical improvement with interleukin-1 blockade in a Spanish cohort. Arthritis Rheumatol (2007) 56(11):3805–13. doi: 10.1002/art.22966 17968944

[18] Poline J Fogel O Pajot C Miceli-Richard C Rybojad M Galeotti C. Early-onset granulomatous arthritis, uveitis and skin rash: characterization of skin involvement in blau syndrome. J Eur Acad Dermatol Venereol JEADV (2020) 34(2):340–8. doi: 10.1111/jdv.15963 31541486

[19] Jha S Mittal S Kumar RR Dhooria A Rawat A Dhir V. An unusual cause of deforming erosive arthritis in an adult. Rheumatol Oxf Engl (2020) 59(3):602. doi: 10.1093/rheumatology/kez312 31377778

[20] Sarens IL Casteels I Anton J Bader-Meunier B Brissaud P Chédeville G. Blau syndrome-associated uveitis: Preliminary results from an international prospective interventional case series. Am J Ophthalmol (2018) 187:158–66. doi: 10.1016/j.ajo.2017.08.017 28887115

[21] Rosé CD Pans S Casteels I Anton J Bader-Meunier B Brissaud P. Blau syndrome: cross-sectional data from a multicentre study of clinical, radiological and functional outcomes. Rheumatol Oxf Engl (2015) 54(6):1008–16. doi: 10.1093/rheumatology/keu437 25416713

[22] Saulsbury FT Wouters CH Martin TM Austin CR Doyle TM Goodwin KA. Incomplete penetrance of the NOD2 E383K substitution among members of a pediatric granulomatous arthritis pedigree. Arthritis Rheum (2009) 60(6):1804–6. doi: 10.1002/art.24532 19479836

